# Virtual Footprints Can Improve Walking Performance in People With Parkinson's Disease

**DOI:** 10.3389/fneur.2018.00681

**Published:** 2018-08-17

**Authors:** Luis I. Gómez-Jordana, James Stafford, C. (Lieke) E. Peper, Cathy M. Craig

**Affiliations:** ^1^Department of Human Movement Sciences, Faculty of Behavioral and Movement Sciences, Vrije Universiteit Amsterdam, Amsterdam, Netherlands; ^2^School of Psychology, Queens University Belfast, Belfast, United Kingdom; ^3^INCISIV Ltd., Belfast, United Kingdom

**Keywords:** Parkinson's disease, visual cue, virtual reality, action-relevant cue, virtual cues

## Abstract

In Parkinson's disease (PD) self-directed movement, such as walking, is often found to be impaired while goal directed movement, such as catching a ball, stays relatively unaltered. This dichotomy is most clearly observed when sensory cueing techniques are used to deliver patterns of sound and/or light which in turn act as an external guide that improves gait performance. In this study we developed visual cues that could be presented in an immersive, interactive virtual reality (VR) environment. By controlling how the visual cues (black footprints) were presented, we created different forms of spatial and temporal information. By presenting the black footprints at a pre-specified distance apart we could recreate different step lengths (spatial cues) and by controlling when the black footprints changed color to red, we could convey information about the timing of the foot placement (temporal cues). A group of healthy controls (HC; *N* = 10) and a group of idiopathic PD patients (PD, *N* = 12) were asked to walk using visual cues that were tailored to their own gait performance [two spatial conditions (115% [N] and 130% [L] of an individual's baseline step length) and three different temporal conditions (spatial only condition [NT], 100 and 125% baseline step cadence)]. Both groups were found to be able to match their gait performance (step length and step cadence) to the information presented in all the visual cue conditions apart from the 125% step cadence conditions. In all conditions the PD group showed reduced levels of gait variability (*p* < 0.05) while the HC group did not decrease. For step velocity there was a significant increase in the temporal conditions, the spatial conditions and of the interaction between the two for both groups of participants (*p* < 0.05). The coefficient of variation of step length, cadence, and velocity were all significantly reduced for the PD group compared to the HC group. In conclusion, our results show how virtual footsteps presented in an immersive, interactive VR environment can significantly improve gait performance in participants with Parkinson's disease.

## Introduction

The loss of dopamine-generating neurons in the basal-ganglia as well as dysfunctional activation of the supplementary motor area, anterior cingulate cortex, and left putamen lead to problems with self-paced movement in people suffering from Parkinson Disease (PD) (1). Although both automated and self-paced movements are impaired by the disease, the ability to control goal-directed or externally cued movements stays relatively unaltered (2, 3). This phenomenon is often known as “kinesia paradoxical” (4) and is demonstrated in studies where self-paced movements, such as walking (5, 6) or reaching (7, 8), improve dramatically when relevant external sensory information is made available.

A nice example of this phenomenon is presented in a study by Asmus et al. (9), who show how a patient suffering from Parkinson's can use the information provided by a ball on the end of a string to move better. The research shows how the moving ball provides an external reference frame onto which the patient can couple his/her movements. This finding suggests that the neural mechanisms behind the control of self-paced movement, where there is no external stimulus or reference to help guide the movement (e.g., walking), are different to the control of movement when external stimuli (e.g., a ball or a metronome) are present (4). Furthermore, it suggests that in these instances where sensory information is available (e.g., visual or auditory) there is a change in the neural mechanisms employed to control movement, effectively bypassing defective basal ganglia circuitry (10).

Sensory cueing is related to the provision of either spatial cues, that give information about where movements should be guided, or temporal cues, that give information about a rhythm (4). The advantages of spatial cues are usually related to an improvement in step length and a reduction in step length variability (10–14). By contrast, the delivery of temporal cues has been shown to improve cadence (i.e., faster walking) and reduce variability (15–17). Traditionally cues have been understood as discrete events that help direct the focus of attention to specific processes that control gait dynamics (18). For this reason, gait cues are usually kept as simple as possible, with cadence being cued through sound using a metronome (19), and step length being cued visually using stripes on the floor, presented perpendicular to the walking direction.

However, our perceptual experience is not a succession of discrete events, but rather a continuous dynamic flow, populated by meaningful happenings (20). In this framework perception can be conceived as the pickup of affordances, understood as possibilities for action (21). If we think of sensory cues as affordances, it is relevant to study how events are specified perceptually, the action-possibilities that are afforded by the cue, and the capabilities of the perceiver to detect and act upon such an event (22). For example, when walking to auditory cues, improvements in the gait of PD patients is directly influenced by the specific nature of the auditory information presented (23). In Young et al.'s (23) study, PD patients walked to the guide of a metronome (containing only temporal information) or to the sound of footsteps over gravel [containing both spatial and temporal information; see (24) for further explanation]. The footsteps were not only able to improve step cadence and reduce step cadence variability in PD patients (as was found with the metronome), but also produced significant changes in step length and step length variability (which was not found for the metronome condition).

Unfortunately, in the case of visual cues, little or no attention has been directed to the nature of the information being delivered through the cues. This study will address the need to understand the type of information being delivered through the visual cues and assess whether patients are able to tune into this information and use it to improve the dynamics of the gait cycle. If we look at the information delivered in visual cues, most studies have focused only on spatial cues that influence step length, such as lines presented perpendicular to the walking direction of the participant (10, 11, 13, 14, 17). This contrasts sharply with the auditory cues used by Young et al. (23), where the sound of footsteps over gravel dynamically convey both the spatial and temporal properties of the action being performed by the walker. This additional information presented in the auditory cues has been defined by Young et al. (23) as “action-relevant” and has been found to be more effective at reducing gait variability (23, 25) and instances and duration of FOG (4) when compared to a traditional metronome. The authors of these studies argued that these improvements may be due to the putative function of “sensory-motor” neurons (23, 24), also known as the mirror neuron system. It is possible that in cueing conditions that are action relevant, this neural system is activated by the perceptual information conveying the spatial-temporal characteristics of the footsteps that generated the sounds.

The aim of the present study was to develop an action-relevant visual cue and test its efficacy using immersive, interactive virtual reality (VR). Although action-relevant cues have been shown to be an effective cueing method when presented in the auditory domain, there is no study that has tested their efficacy when presented in the visual domain. In this study, different types of visual cues were developed and tested in an immersive, interactive VR environment that looked like a regular hallway. In this way, we ensured high ecological validity, while maintaining precise control over the experimental conditions (26). Taking the perspective that perception is scaled to the action capabilities of the perceiver (27–30), the cues were adapted to each participant with respect to their own baseline gait measures that were taken at the start of the session. Furthermore, the cues were manipulated in order to convey different spatial and temporal information.

It is hypothesized that participants will be able to tune into the two types of information (spatial and temporal) delivered by the visual cues, resulting in improvements in specific gait parameters, namely step length, step cadence, step velocity, and the variability of each of these gait parameters. In particular, the footprints with only spatial information are expected to improve step length, step velocity and the variability of these parameters. The footprints that deliver both spatial and temporal information combined are anticipated to also reduce step cadence variability and increase step cadence as a function of the rhythm presented in the cues. Furthermore, the improvements in step length and step velocity are predicted to vary between the cues as a function of the different forms of spatial information presented in the cue conditions. Finally, as PD patients are expected to have larger gait variability in their baseline measures, the decreases in variability are expected to be larger in this group compared to the Healthy Control (HC) group (23).

## Methods

### Participants

Two groups of participants were recruited: one group of Healthy Controls (HC; *N* = 10; mean age = 63.0 years; *SD* = 8.6 years, 6 females, 4 males), and a group of idiopathic PD patients (PD; *N* = 12; mean age = 65.3 years; *SD* = 7.6 years, 6 females, 6 males). The PD group was recruited through a newsletter distributed to members of Parkinson's UK. Motor disability was assessed using part III of the MDS-UPDRS questionnaire (31). This test consists of 33 items, each of which is scored from 0 to 4, with higher scores indicating more severe symptoms. “The freezing of gait questionnaire” [FOGQ; (32)] was used to asses if participants had experienced FOG consistently in the week prior to the experiment. A mean score >2 indicated that this was the case. The results of this test are presented in Table 1, along with demographic information about the PD participants. Due to ethical considerations we were not able to interfere with the medication regimes of the PD patients, therefore some patients were tested in an OFF state while others in an ON state. One healthy adult withdrew from the experiment before completing the study. The data from this participant has been excluded from the analysis. This participant was replaced by another healthy control (HC). The study was approved by the University ethics committee (PREC-31-2016-17). All participants gave written informed consent and sought permission to participate from their medical practitioner. None of the participants had any known cognitive impairments (note no cognitive tests were administered).

**Table 1:** Demographic information about the PD participants.

**Participants**	**Age (years)**	**Gender**	**Years from diagnosis**	**Clinical state**	**UPDRS part III**	**FOG-Q**
PD1	61	Female	7	ON	10	0.33 (0.51)
PD2	77	Male	6	OFF	29	0.66 (1.06)
PD3	64	Female	2	ON	18	0.33 (0.52)
PD4	66	Female	4	ON	37	0.5 (0.55)
PD5	50	Male	2	ON	23	0.66 (1.03)
PD6	58	Male	7	ON	27	0.66 (1.03)
PD7	68	Female	6	OFF	35	3.00 (0.63)
PD8	76	Male	5	ON	28	3.16 (0.43)
PD9	59	Female	4	ON	36	2.66 (0.81)
PD10	69	Male	6	OFF	41	3.33 (0.51)
PD11	70	Female	10	ON	43	0.66 (1.06)
PD12	66	Male	4	ON	37	0.5 (0.55)

*The clinical state is related to the effect of the medication: ON, responding well to medication; OFF, not responding to medication*.

### Immersive, interactive virtual reality

A virtual environment (representing a hallway) was presented through a virtual reality head-set (Oculus Rift, DK2, Irvine, California, USA). The screen had a resolution of 1,920 × 1,080, had a field of view of 100 degrees and was updated 75 times per second. Intersense IS900 (InterSense Inc., Bedford, Massachusetts, USA) tracking system was used instead of the Oculus tracking to allow participants the freedom to walk up and down the virtual hallway. In this way we were able to facilitate a one to one mapping between participant movement in the real world and movement in the virtual world. Both head orientation and position were tracked and updated in the virtual environment at 120 Hz. This in turn updated the participant's viewpoint but also served as a measure of how the participant moved through the environment. The tracked space was 12 m long by 5 m wide.

### Walking metrics

Although the information provided by the Intersense 900 head tracker measured participant movement through the environment, more detailed information about the participant's walking characteristics was also required. Participants had a rigid body containing three reflective markers attached to each foot that allowed us to capture this data. A set of 12 Qualisys infrared motion capture cameras (Qualisys Ltd., Göteborg, Sweden) recorded the movement of these reflective markers at 100 Hz. Furthermore, to allow participants to see their own feet in the virtual environment, data were streamed in real-time from the infrared motion capture system into the virtual environment (Qualisys Unity SDK), with a sampling frequency of 30 HZ. The position of the two rigid bodies was automatically detected by the system, and was used to control the position and orientation of virtual depictions of the feet (in the form of two blue cuboid shaped boxes). The virtual representation of the foot allowed participants to see where they were stepping, increasing the levels of behavioral realism (33).

### Visual cues

**Figure 1:**
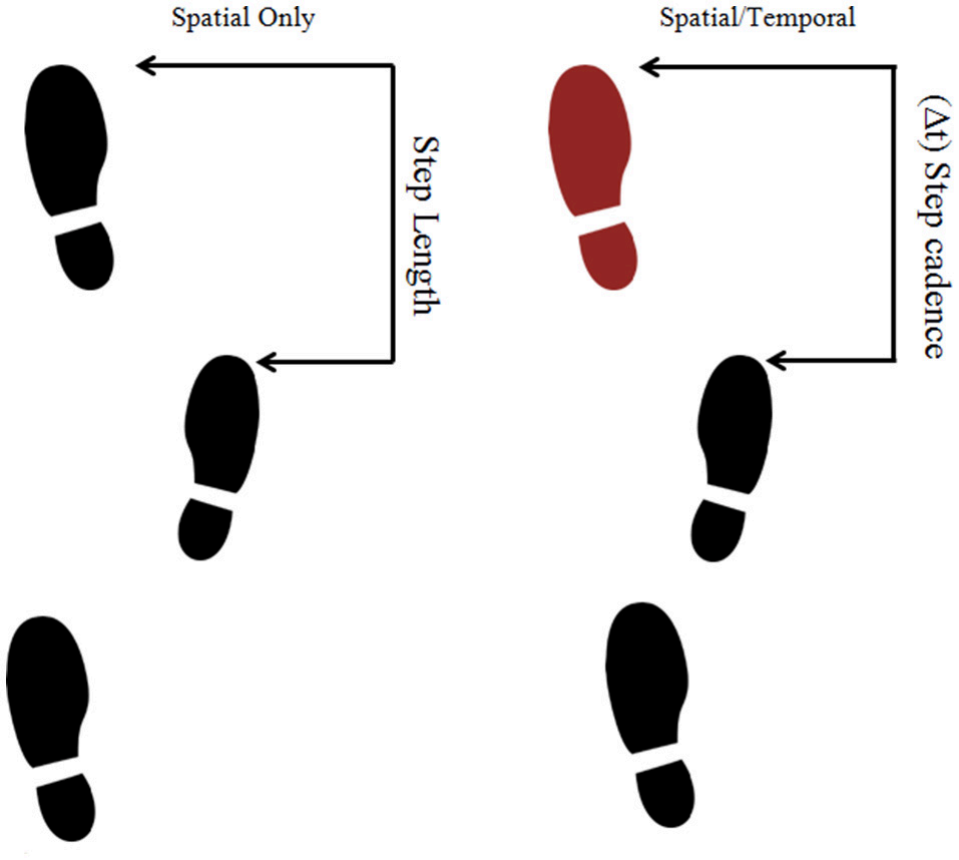
Examples of the presentation of three footprints as if they were the cues used in the experiment. The left figure corresponds to a spatial only cue while the right figure corresponds to a spatio-temporal cue.

Two different types of visual cues were created: one representing step length (spatial information) and the other representing step-length and cadence (spatial-temporal information). To increase the ecological validity the cues were presented as a sequence of black footprints (25.5 cm long and 12.75 cm wide—equivalent to European shoe size 40) projected onto the virtual floor. The difference between successive footprints represented step length (spatial information), whilst the rhythmic lighting up of a footprint (changing color from black to red) represented the cadence (temporal information; see Figure 1). Cues were personalized for each individual according to their baseline gait characteristics (see Procedure for more details). Two different step lengths were used (115% of baseline [“normal;” N]), and (130% of baseline step length [“long;” L]) and these were crossed with three different types of temporal cues (No temporal information [NT], 100% of baseline cadence [100%], and 125% of baseline cadence [125%]). This gave rise to six different cue conditions, two spatial only conditions (N-NT and L-NT) and four spatio-temporal conditions (N-100%, N-125%, L-100%, and L-125%).

### Virtual hallways

**Figure 2:**
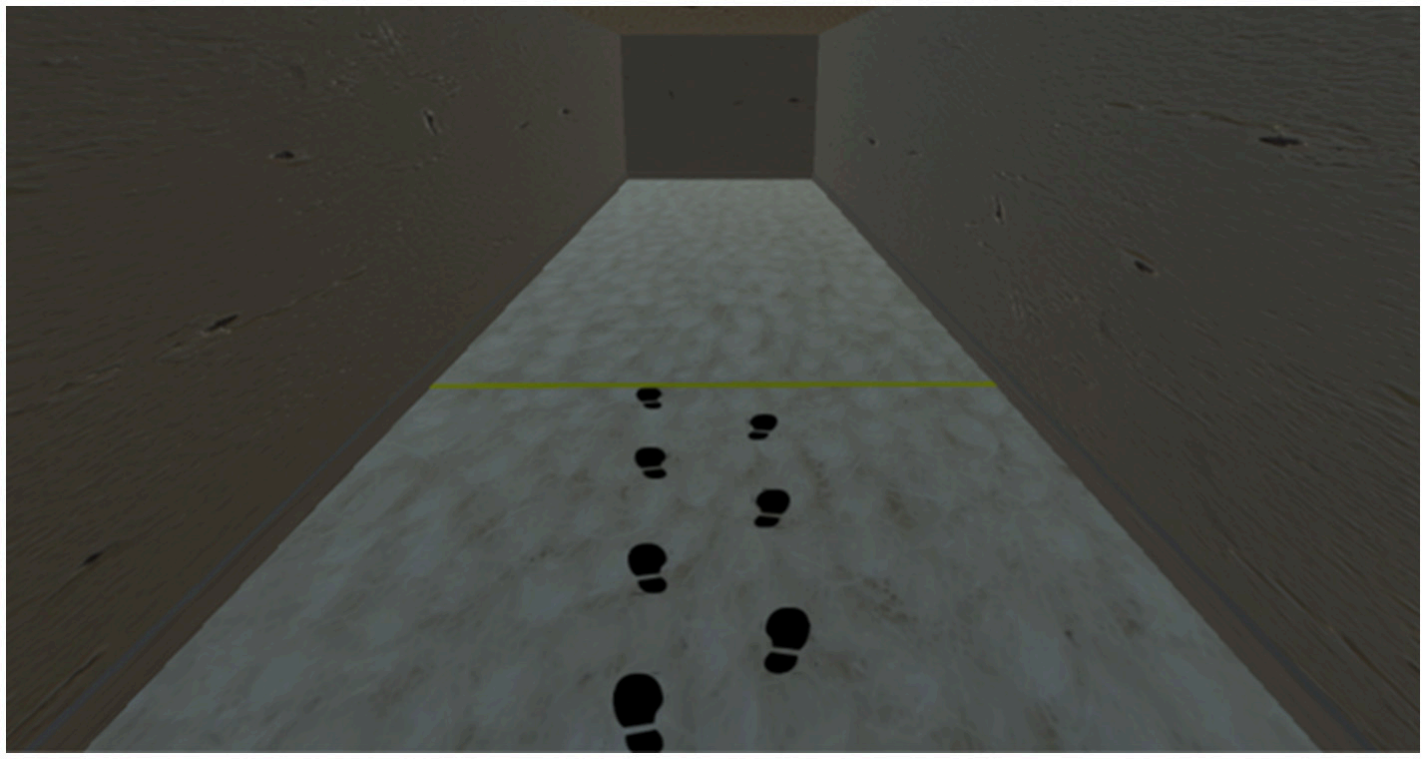
Screenshot of the participant's view of the hallway during a practice trial that included a spatial only cue.

The game engine software Unity (version 5.4.1f1) was used to construct the virtual environment. It consisted of a virtual hallway 20 m long and 2.5 m high (see Figure 2). A clay texture was added to the walls and ceiling, while a white carpet texture was added to the floor to increase the level of picture realism of the virtual environment. For each participant the width of the hallway was personalized to represent five shoulder widths. This parameter was inputted at the start of the experiment. Participants walked back and forth between red and yellow lines that were placed 6.5 m apart. In the even trials participants walked toward the red line while in the odd trials the participants walked in the opposite direction toward the yellow line.

### Procedure

At the start of the experiment, the participant's shoulder width was measured (using a tape measure) and entered into Unity. This allows the program to automatically scale the width of the virtual hallway to the participant's own bodily proportions. Two experimenters were always present. One experimenter controlled the virtual environment and the motion capture system while the other experimenter walked next to the participant, holding the cable that connected the headset to the computer, ensuring the participant's safety. Firstly, participants completed four familiarization trials to get used to walking in the VR system. Then, each participant performed eight walking trials in the virtual hallway, without cues. The recorded data from the last four walks were used to calculate a baseline measure of step length and step cadence (see Table 2 for the baseline results). These measures were used to construct personalized cue parameters (step length and cadence) for each participant. After this, participants were given a familiarization period where they walked eight more times guided by visual cues containing either spatial (step length) or spatio-temporal (cadence and step-length) information. These familiarization cues had slightly different spatial and temporal parameters than the ones used in the experimental trials and were intended to familiarize the participant with the presence of the visual cues. Each trial consisted of a 6.5 m walk toward the end line. The trial ended once they reached the line indicating the end of the trial and participants, with the help of the experimenter, were asked to turn around and face the opposite direction ready for the next trial. Once they had turned around and indicated they were ready, a new trial started in which they walked toward the other end line. At the end of the block of practice trials, participants were allowed to rest for 2 min.

**Table 2:** Table containing the results for the six different gait parameters in the baseline block.

**Gait parameter**	**Group**	**Baseline result**
Step length	HC	0.61 m (0.11 m)
	PD	0.50 m (0.9 m)
Step cadence	HC	1.77 Hz (0.21 Hz)
	PD	1.65 Hz (0.29 Hz)
Step velocity	HC	1.08 m/s (0.21 m/s)
	PD	0.85 m/s (0.24 m/s)
Step length CV	HC	0.06 (0.03)
	PD	0.17 (0.10)
Step cadence CV	HC	0.07 (0.03)
	PD	0.16 (0.14)
Step velocity CV	HC	0.09 (0.04)
	PD	0.21 (0.09)

*The results are presented as the mean values (standard deviations in brackets)*.

The six different cue conditions (two spatial only and four spatio-temporal) were pseudo-randomly presented. In the spatio-temporal conditions, one footprint at a time changed color from black to red to specify the cadence. This rhythmic left-right footprint color change was designed to mimic the walking characteristics of an invisible person walking in front of the participant. Participants were instructed to walk on the footprints on the floor, and to try to match their rhythm to the rhythm imposed by the color change, if such a change was present. Video S1 includes what a participant saw in the VR hallway as they completed one trial of each block. Before the start of the block of trials participants were informed of the type of cue (spatial or spatio-temporal) that they were going to see. Each cue was presented in one of six walking trial blocks with the type of cue in each block being determined randomly. Once participants reached the line indicating the end of the trial, participants with the help of the experimenter, were asked to turn around and face the opposite direction ready for the start of the next trial. This method for collecting walking data halved the amount of walking a participant had to perform thereby minimizing any fatigue that could be associated with repeatedly walking back toward the same starting position. Each block consisted of eight trials, and for each block of eight trials participants were allowed to rest for 2 min. The procedure lasted about 40 min in total.

### Gait analysis

The Qualisys data were analyzed using custom-made Matlab (Matlab, 2016b; Mathworks, Inc., Natick, Massachusetts, USA) routines. The gait data was low-pass filtered (Recursive Butterworth; 2nd order; cut-off frequency: 10 Hz), and then heel strikes were marked automatically based on the moments at which the marker nearest to the heel reached zero velocity in a vertical direction. These heel strikes were used to calculate step length, cadence, and step velocity. Step length was formalized as the distance between two successive heel strikes in the direction of walking. Step cadence was defined as the number of steps taken per second. Step velocity was calculated by dividing step length by the time it took to complete. Also, for each trial the mean and coefficient of variation (CV, standard deviation divided by the mean) of these parameters was also determined.

As the objective of the study was to test if the visual cues were able to induce changes in gait dynamics, all gait parameters were expressed as a ratio with respect to the corresponding baseline measurement obtained for each individual participant. This ratio can be understood as expressing the proportion of change from the baseline measures. Thus, values >1 indicate gait performance that was better than baseline, while values < 1 indicate gait performance that was worse than baseline.

### Statistical analysis

The statistical analyses were carried out using Rstudio (Rstudio 1.138; RStudio, Inc., Boston, MA). Firstly, we conducted one-sample *t*-tests on the individual results of each group for step length and step cadence, comparing mean results to the step length and step cadence imposed by the visual cue during the corresponding block of trials. Step length results are presented in meters while step cadence is presented in Hertz. The objective of these comparisons was to see if participants were adhering to the step length and cadence imposed by each cue. Secondly, to determine whether the gait parameters were significantly affected by the visual cues, we examined the 95%-confidence intervals for each block of trials for each group of participants. If 1 was not included in the confidence intervals, this meant that they were significantly different to the baseline results. This analysis allowed us to see if the cues produced improvements in the gait parameters for all of the participants. Thirdly, we conducted three-way mixed ANOVAs on the results for all of the gait parameters with the between-subjects factor being Group (HC, PD) and the within-subjects factors being Spatial information (N, L) and Temporal information (NT, 100%, and 125%). The results were considered significant when *p* < 0.05. *Post-hoc* comparisons were based on simple effects analysis (34) and (if required) pairwise tests with Bonferroni corrections were used. Results are presented as the mean plus or minus one standard deviation. Effect size was presented using ω_*p*_^2^, which is believed to provide a better estimate than η_*p*_^2^ (35). This statistic can take values from 0 to 1, with higher values indicating higher size effects. Because R does not include a built-in formula that calculates ω_*p*_^2^, a custom-made function for calculating ω_*p*_^2^ was created using the formula in Olejnik and Algina (36).

## Results

### Task adherence

The *t*-tests showed that the HC actual step length (N: 0.69 ± 0.10 m; L: 0.77 ± 0.12 m) did not differ significantly from the imposed values (N: 0.70 m; L: 0.79 m) in any of the conditions. The *t*-tests also showed that the actual step length for PD patients (*N*: 0.56 ± 0.10 m; L: 0.62 ± 0.11 m) did not differ from the imposed step length in any of the conditions (*N*: 0.57 m; L: 0.65 m). With respect to step cadence, the *t*-tests revealed that both groups adhered (HC: 1.81 ± 0.22 Hz; PD: 1.69 ± 0.31 Hz) to the 100% condition (HC: 1.77 Hz; PD: 1.59 Hz), but neither of the groups fully adapted to the 125% condition [HC: *t*_(19)_ = −3.84, *p* = 0.001, 1.94 ± 0.34 Hz; PD: *t*_(23)_ = −3.02, *p* = 0.006, 1.80 ± 0.30 Hz], indicating that the imposed cadence (HC: 2.21 Hz; PD: 1.99 Hz) in this condition was too fast to use as a guide to improve gait performance.

### Changes relative to baseline

**Figure 3:**
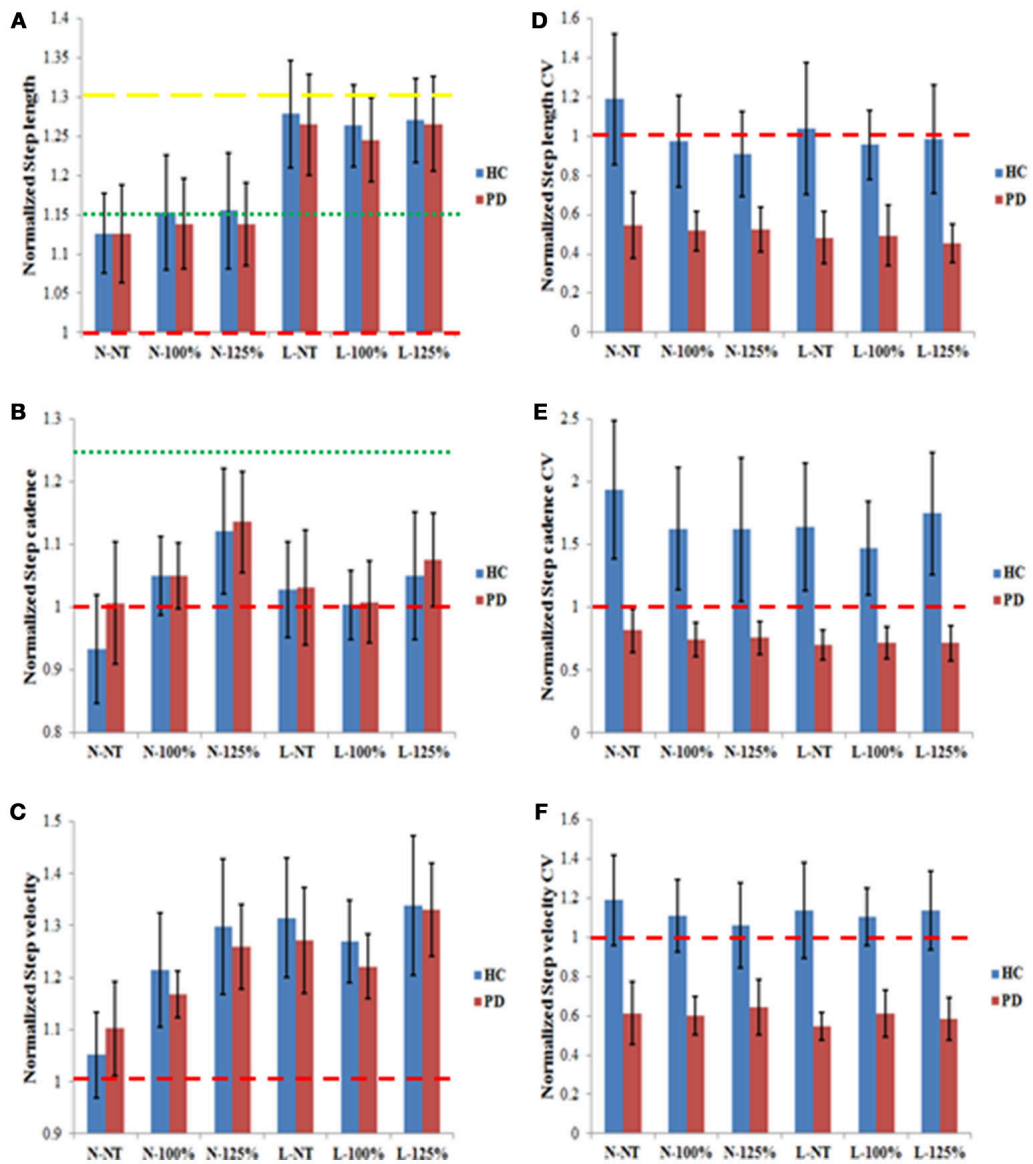
Normalized results for step length **(A)**, step cadence **(B)**, step velocity **(C)**, step length CV **(D)**, step cadence CV **(E)**, and step velocity CV **(F)** for the PD and the HC groups in the six different experimental conditions. The results are presented as normalized results calculated as the values obtained divided by the corresponding individual baseline values. Values >1 indicate an increase relative to baseline; values < 1 indicate a decrease relative to baseline. The red dashed lines in all graphs represent the value that would indicate no difference from baseline measures (1). In **(A)** the green and yellow dashed lines represent the step length prescribed in the N and L conditions, respectively. In **(B)** the dashed red and the green lines represent the step cadence prescribed in the 100 and 125% conditions, respectively. The error bars represent the confidence intervals (95%) of the mean.

As can be seen from the confidence intervals shown in Figure 3A, all cueing conditions were effective in increasing step length in both groups. Step cadence (Figure 3B) was significantly increased in the N-125% condition, in both groups. Note that for this variable no increase was expected for the 100% conditions, as they prescribed the same cadence established in the baseline trials. For step velocity (Figure 3C) increased values were obtained for all conditions in the PD group, while in the HC group only the N-NT condition did not induce a significant increase in velocity which would be expected given the closeness of the cue to the baseline condition. The coefficient of variation (CV) for step length (Figure 3D), step cadence (Figure 3E), and step velocity (Figure 3F) in all the conditions produced improvements (i.e., lower values) in the PD group, but none of the conditions produced improvements in the HC group. In fact, in the HC group all the conditions, except for N-NT had a larger coefficient of variation (CV) for step cadence.

### Comparison between cueing conditions

The results of the ANOVA for step length (see Figure 3A) showed a significant main effect for Spatial information [*F*_(1, 21)_ = 49.12, *p* < 0.001, ω_*p*_^2^ = 0.192], indicating a greater improvement in performance in the L condition (1.26 ± 0.10) compared to the N condition (1.14 ± 0.10). The ANOVA for step cadence (see Figure 3B) yielded a significant main effect of Temporal information [*F*_(2, 21)_ = 5.79, *p* = 0.004, ω_*p*_^2^ = 0.042] with *post-hoc* analysis revealing that the fastest temporal condition (125%) yielded a larger increase in step cadence (1.10 ± 0.15) than the two other conditions (NT: 1.00 ± 0.15; 100%: 1.03 ± 0.10). The ANOVA for step velocity (see Figure 3C) revealed a significant main effect for Spatial information [*F*_(1, 21)_ = 15.00, *p* < 0.001, ω_*p*_^2^ = 0.085], indicating that the participants moved faster in the L condition (1.29 ± 0.16) than in the N condition (1.18 ± 0.15). The effect of Temporal information was also significant [*F*_(2, 21)_ = 6.76, *p* = 0.002, ω_*p*_^2^ = 0.053] with participants moving significantly quicker in the fastest condition (125%: 1.31 ± 0.18) compared to the two other conditions (NT: 1.18 ± 0.16; 100%: 1.22 ± 0.12; *p* < 0.05). Finally, the interaction between the Spatial and Temporal information was also significant [*F*_(2, 21)_ = 3.53, *p* = 0.033, ω_*p*_^2^ = 0.016]. *Post-hoc* analysis revealed that for the spatial condition *N*, the temporal condition 125% induced significantly larger improvements (1.28 ± 0.18) than either of the two other temporal conditions (NT: 1.08 ± 0.15, 100%: 1.19 ± 0.13). The Temporal factor did not significantly affect the degree of improvement in the spatial condition L (NT: 1.29 ± 0.18, 100%: 1.24 ± 0.12, 125%: 1.33 ± 0.19; *p* > 0.05), whereas the main effect of Spatial information was observed for all three temporal conditions (*p* < 0.05).

The ANOVAs for the three CV measures (see Figures 3D–F) only showed significant effects for group [step length CV: *F*_(1, 21)_ = 73.71, *p* < 0.001, ω_*p*_^2^ = 0.313; step cadence CV: *F*_(1, 21)_ = 56.15, *p* < 0.001, ω_*p*_^2^ = 0.278; step velocity CV: *F*_(1, 21)_ = 119.84, *p* < 0.001, ω_*p*_^2^ = 0.543] with PD participants (step length CV: 0.50 ± 0.23; step cadence CV: 0.74 ± 0.26; step velocity CV: 0.60 ± 0.21) showing significant improvements compared to the HC group (step length CV: 1.01 ± 0.42; step cadence CV: 1.67 ± 0.95; step velocity CV: 1.12 ± 0.33; *p* < 0.05).

## Discussion

In this experiment, we examined whether action-relevant visual cues had a positive effect on gait parameters in PD patients. We compared spatial and spatio-temporal cueing conditions, which all involved the presentation of footsteps in a VR environment. As a reference, we also examined the effects of the cues on a healthy control (HC) group. The analysis focused on two questions: (i) Did the visual cues improve the gait parameters relative to our baseline measurements without cues in participants with Parkinson's, and (ii) Did the different cueing conditions yield different levels of improvement on chosen gait parameters? The extent to which our results answer these questions is addressed below.

To determine (within 95% confidence intervals) if the cues significantly improved gait parameters (step length, step cadence, step velocity, and their CVs), we compared whether the ratio between actual performance and baseline performance, differed significantly from 1. This analysis showed clear improvements in the PD group for all cueing conditions, in particular with respect to step length, step velocity, and overall gait variability. For step cadence, improvement was only observed for the N-125%, i.e., the condition that required a faster cadence in combination with a normal step length. Although for the HC group most cueing conditions also induced improvements in step length, and step velocity, they did not yield any improvements in gait variability, which may reflect a ceiling effect given the low variability of values at baseline (see Table 2).

Using ANOVAs we also compared the influence of the six cueing conditions, to determine their relative effectiveness on gait performance. As expected the spatial information impacted on step length, temporal information influenced step cadence, while both factors influenced step velocity. The two groups (PD and HC) did not appear to differ in this respect. As we examined the ratio between the actual performance and the baseline performance, this implies that both groups showed statistically equivalent degrees of improvement in the examined gait parameters. The ANOVAs on the variability measures (CVs), however, did not show any differences between the cueing types, but yielded a significant difference between the two groups. Overall, the PD group showed stronger improvements than the HC group, which is consistent with the potential ceiling effect mentioned above.

Together, these results indicate that our visual cues were effective in improving gait parameters in PD. The spatial and temporal factors specifically improved the mean values of the associated gait characteristics. As spatial information was included in all cueing conditions (given the presentation of the footsteps), all conditions resulted in improved step length and velocity. Combining this information with temporal information, yielded improvements in cadence as well, which was revealed by the significant difference between the 125% condition and the 100% and NT conditions. Since the 100% condition corresponded to the baseline cadence obtained for each individual, the observed absence of a difference between the latter two conditions was in accordance with our expectations. Moreover, for the PD patients, all cueing types resulted in a reduction in all gait variability measures.

The participants were able to re-enact the information contained in all cueing conditions, except for the temporal information contained in the 125% temporal condition that appeared to be too fast for the participants. Nevertheless, this condition yielded significant improvements in step cadence and velocity (for both groups), as well as in all variability measures in the PD group. Moreover, the improvements for step cadence and step velocity were greater for this condition (125%) compared to the other two temporal conditions when presented with the spatial condition N. The absence of an effect in the spatial condition L, may indicate that this combination of higher cadence and longer step length was too challenging.

A common way to interpret the effectiveness of visual cues is assuming that they help to direct attention to gait characteristics that are impaired (10, 18). Visual cues would serve as a way of correctly activating the motor areas related to walking (14) by centering the focus of attention on the part of gait that is impaired. This hypothesis was supported by studies that showed that the advantage experienced while walking using visual cues gets aborted when performing a dual task (13, 14).

In the present study we did not address the associated cognitive processes, but focused on the functional characteristics of the task, in particular, the informational constrains of the task (4, 8, 19, 23–25). In doing so, we improved the action-relevance of our cues, compared to the more commonly used perpendicular lines. This improvement resides in the incorporation of the alternating lateral symmetry of gait that was reflected in the virtual “footsteps” [similar to the stepping stones used by (37)] and combining it with cadence information. In addition, we scaled our cues to the action capabilities of our participants, so that any increase had functional specificity.

To present these cues we used an immersive, interactive VR environment. This set-up allowed us to reliably deliver cues that were attuned to each individual in a quick and safe manner. Although the use of VR may be practical in experimental research and rehabilitation (38), it remains a challenge to create effective cueing interventions when patients are experiencing debilitating symptoms in real life. One promising technological development in this regard is that of augmented reality (39). As these glasses allow for the delivery of visual information that is superimposed on the visual field of the patient, they open up new possibilities to provide relevant, ecologically valid information, while the patient is walking naturally in the real world. Going forward it will be important to consider the most effective task-relevant cues to alleviate other coordination problems such as turning, initiating movement and crossing doorways.

In summary, our action-relevant visual cues improved mean gait parameters in both the PD and HC group. Spatial information (present in all cues) improved step length and velocity. Combining this information with temporal information, yielded improvements in cadence as well. Moreover, for the PD patients, all gait variability measures were significantly reduced in response to all cueing types. Together these results indicate that although spatial visual cues may improve Parkinsonian gait, the effects of visual cueing can be expanded to the domain of step cadence by enriching the cues with temporal information as well. The action relevance of such spatio-temporal cues is evident, as gait is inherently a spatio-temporal activity.

## Ethics statement

This study was carried out in accordance with the recommendations of the Queen's University Belfast Policy and Principles on the Ethical Approval of Research, Queen's University of Belfast ethics committee. The protocol was approved by the Queen's University of Belfast ethics committee. All subjects gave written informed consent in accordance with the Declaration of Helsinki.

## Author contributions

LG-J: This research was designed, and developed as the Master Thesis of this author. This author was involved in all the phases, designing the experiment, preparing the virtual environments, recruiting the patients, running the experimental protocols, processing the data, analyzing the data, and writing this article. JS: He helped with the recruitment of the patients. Furthermore, he was the second experimenter that was always present when the experiment was being run. CP: She was the first supervisor of the master thesis. She assisted in the design, as well as in the writing and the analysis of the data. CC: She was the second supervisor of the master thesis. She assisted in the implementation of the design, the recruitment of the patients, as well as in the preparation of the virtual environment and in the process of writing this article.

### Conflict of interest statement

The authors declare that the research was conducted in the absence of any commercial or financial relationships that could be construed as a potential conflict of interest.

## Supplementary material

Video S1 Video shows what it was like inside the VR environment when a participant (Healthy Control) completed all 6 conditions.

## References

[1] Jahanshahi M Jenkins IH Brown RG Marsden CD Passingham RE Brooks DJ. Self-initiated versus externally triggered movements. Brain (1995) 118:913–33. 10.1093/brain/118.4.913 7655888

[2] Redgrave P Rodriguez M Smith Y Rodriguez-Oroz MC Lehericy S Bergman H. Goal-directed and habitual control in the basal ganglia: implications for Parkinson's disease. Nat Rev Neurosci. (2010) 11:760–72. 10.1038/nrn2915 20944662 PMC3124757

[3] Torres EB. Two classes of movements in motor control. Exp Brain Res. (2011) 215:269–83. 10.1007/s00221-011-2892-8 22038712

[4] Young WR Shreve L Quinn EJ Craig C Bronte-Stewart H. Auditory cueing in Parkinson's patients with freezing of gait. What matters most: action-relevance or cue-continuity? Neuropsychologia (2016) 87:54–62. 10.1016/j.neuropsychologia.2016.04.034 27163397

[5] Rubinstein TC Giladi N Hausdorff JM. The power of cueing to circumvent dopamine deficits: a review of physical therapy treatment of gait disturbances in Parkinson's disease. Mov Disord. (2002) 17:1148–60. 10.1002/mds.10259 12465051

[6] Lim I van Wegen E de Goede C Deutekom M Nieuwboer A Willems A. Effects of external rhythmical cueing on gait in patients with Parkinson's disease: a systematic review. Clin Rehab. (2005) 19:695–713. 10.1191/0269215505cr906oa 16250189

[7] Majsak MJ Kaminski T Gentile AM Flanagan JR. The reaching movements of patients with Parkinson's disease under self-determined maximal speed and visually cued conditions. Brain (1998) 121:755–66. 10.1093/brain/121.4.755 9577399

[8] Bienkiewicz MM Rodger MW Young WR Craig CM. Time to get a move on: overcoming bradykinetic movement in Parkinson's disease with artificial sensory guidance generated from biological motion. Behav Brain Res. (2013) 253:113–20. 10.1016/j.bbr.2013.07.003 23838076

[9] Asmus F Huber H Gasser T Schöls L. Kick and rush: paradoxical kinesia in Parkinson disease. Neurology (2008) 73:328–9. 10.1212/01.wnl.0000324618.88710.30 18725599

[10] Morris ME Iansek R Matyas TA Summers JJ. Stride length regulation in Parkinson's disease: normalization strategies and underlying mechanisms. Brain (1996) 119:551–68. 10.1093/brain/119.2.551 8800948

[11] Azulay JP Mesure S Amblard B Blin O Sangla I Pouget J. Visual control of locomotion in Parkinson's disease. Brain (1999) 122:111–20. 10.1093/brain/122.1.111 10050899

[12] Lewis GN Byblow WD Walt SE. Stride length regulation in Parkinson's disease: the use of extrinsic, visual cues. Brain (2000) 123:2077–90. 10.1093/brain/123.10.2077 11004125

[13] Morris ME Iansek R Matyas TA Summers JJ. Ability to modulate walking cadence remains intact in Parkinson's disease. J Neurol Neurosurg Psychiatry (1994) 57:1532–4. 10.1136/jnnp.57.12.1532 7798986 PMC1073238

[14] Morris ME Iansek R Matyas TA Summers JJ. The pathogenesis of gait hypokinesia in Parkinson's disease. Brain (1994) 117:1169–81. 10.1093/brain/117.5.1169 7953597

[15] Hausdorff JM Lowenthal J Herman T Gruendlinger L Peretz C Giladi N. Rhythmic auditory stimulation modulates gait variability in Parkinson's disease. Eur J Neurosci. (2007) 26:2369–75. 10.1111/j.1460-9568.2007.05810.x 17953624

[16] McIntosh GC Brown SH Rice RR Thaut MH. Rhythmic auditory-motor facilitation of gait patterns in patients with Parkinson's disease. J Neurol Neurosurg Psychiatry (1997) 62:22–6. 10.1136/jnnp.62.1.22 9010395 PMC486690

[17] Suteerawattananon M Morris GS Etnyre BR Jankovic J Protas EJ. Effects of visual and auditory cues on gait in individuals with Parkinson's disease. J Neurolog Sci. (2004) 219:63–9. 10.1016/j.jns.2003.12.007 15050439

[18] Azulay JP Mesure S Blin O Influence of visual cues on gait in Parkinson's disease: contribution to attention or sensory dependence? J Neurol Sci. (2006) 248:192–5. 10.1016/j.jns.2006.05.008 16765379

[19] Rodger M Craig C. Beyond the metronome: auditory events and music may afford more than just interval durations as gait cues in Parkinson's disease. Front Neurosci. (2016) 10:272. 10.3389/fnins.2016.00272 27378841 PMC4906221

[20] Gibson J Events are perceived but time is not. In: Fraser J Lawrence N, editors. The Study of Time, Vol. 2. New York, NY: Springer (1975). p. 295–301.

[21] Gibson JJ The Ecological Approach to Visual Perception: Classic Edition. Boston, MA: Houghton-Mifihin (1979).

[22] Steenson CJ Rodger MW Bringing sounds into use: thinking of sounds as materials and a sketch of auditory affordances. Open Psychol J. (2015) 8:174–82. 10.2174/1874350101508010174

[23] Young WR Rodger MW Craig CM Auditory observation of stepping actions can cue both spatial and temporal components of gait in Parkinson's disease patients. Neuropsychologia (2014) 57:140–53. 10.1016/j.neuropsychologia.2014.03.009 24680722

[24] Young W Rodger M Craig CM. Perceiving and reenacting spatiotemporal characteristics of walking sounds. J Exp Psychol Hum Percept Perform. (2013) 39:464–77. 10.1037/a0029402 22866760

[25] Rodger MW Young WR Craig CM. Synthesis of walking sounds for alleviating gait disturbances in Parkinson's disease. IEEE Trans Neural Syst Rehab Eng. (2014) 22:543–8. 10.1109/TNSRE.2013.2285410 24235275

[26] Loomis JM Blascovich JJ Beall AC. Immersive virtual environment technology as a basic research tool in psychology. Behav Res Meth Instrum Comput. (1999) 31:557–64. 10.3758/BF03200735 10633974

[27] van der Kamp J Savelsbergh GJ Davis WE Body-scaled ratio as a control parameter for prehension in 5-to-9-year-old-children. Dev Psychobiol. (1998) 33:351–61. 10.1002/(SICI)1098-2302(199812)33:4<351::AID-DEV6>3.0.CO;2-P 9846238

[28] Mark LS. Eyeheight-scaled information about affordances: a study of sitting and stair climbing. J Exp Psychol Hum Percept Perform. (1987) 13:361–70. 10.1037/0096-1523.13.3.361 2958585

[29] Warren WH. Perceiving affordances: visual guidance of stair climbing. J Exp Psychol Hum Percept Perform. (1984) 10:683–703. 10.1037/0096-1523.10.5.683 6238127

[30] Warren WH Whang S. Visual guidance of walking through apertures: body-scaled information for affordances. J Exp Psychol Hum Percept Perform. (1987) 13:371382. 10.1037/0096-1523.13.3.371 2958586

[31] Goetz CG Tilley BC Shaftman SR Stebbins GT Fahn S Martinez-Martin P. Movement Disorder Society-sponsored revision of the Unified Parkinson's Disease Rating Scale (MDS-UPDRS): scale presentation and clinimetric testing results. Mov Disord. (2008) 23:2129–70. 10.1002/mds.22340 19025984

[32] Giladi N Shabtai H Simon ES Biran S Tal J Korczyn AD. Construction of freezing of gait questionnaire for patients with Parkinsonism. Parkinsonism Relat Disord. (2000) 6:165–70. 10.1016/S1353-8020(99)00062-0 10817956

[33] Craig C Understanding perception and action in sport: how can virtual reality technology help? Sports Technol. (2014) 6:161–9. 10.1080/19346182.2013.855224

[34] Keppel G Zedeck S Data Analysis for Research Designs. New York, NY: Freeman (1989).

[35] Okada K Is omega squared less biased? A comparison of three major effect size indices in one-way ANOVA. Behaviormetrika (2013) 40:129–47. 10.2333/bhmk.40.129

[36] Olejnik S Algina J. Generalized eta and omega squared statistics: measures of effect size for some common research designs. Psychol Methods (2003) 8:434–47. 10.1037/1082-989X.8.4.434 14664681

[37] Bank PJ Roerdink M Peper CE. Comparing the efficacy of metronome beeps and stepping stones to adjust gait: steps to follow! Exp Brain Res. (2011) 209:159–69. 10.1007/s00221-010-2531-9 21221956 PMC3041909

[38] Schultheis MT Rizzo AA The application of virtual reality technology in rehabilitation. Rehab Psychol. (2001) 46:296–312. 10.1037/0090-5550.46.3.296

[39] Janssen S Bolte B Nonnekes J Bittner M Bloem BR Heida T. Usability of three-dimensional augmented visual cues delivered by smart glasses on (freezing of) gait in Parkinson's disease. Front Neurol. (2017) 8:279. 10.3389/fneur.2017.00279 28659862 PMC5468397

